# IGNITE network: Response of patients to genomic medicine interventions

**DOI:** 10.1002/mgg3.636

**Published:** 2019-03-20

**Authors:** Lori A. Orlando, Corrine Voils, Carol R. Horowitz, Rachel A. Myers, Meghan J. Arwood, Emily J. Cicali, Caitrin W. McDonough, Toni I. Pollin, Yue Guan, Kenneth D. Levy, Andrea Ramirez, Alexandra Quittner, Ebony B. Madden

**Affiliations:** ^1^ Department of Medicine and the Center for Applied Genomics and Precision Medicine Duke University Durham North Carolina; ^2^ School of Medicine & Public Health William S Middleton Memorial Veterans Hospital, University of Wisconsin Madison Wisconsin; ^3^ Department of Population Health Sciences and Policy and the Center for Health Equity and Community Engaged Research Icahn School of Medicine at Mount Sinai New York New York; ^4^ Department of Pharmacotherapy and Translational Research and Center for Pharmacogenomics, College of Pharmacy University of Florida Gainesville Florida; ^5^ Department of Medicine University of Maryland Baltimore Maryland; ^6^ Division of Clinical Pharmacology, Department of Medicine Indiana University School of Medicine Indianapolis Indiana; ^7^ Department of Medicine Vanderbilt University Nashville Tennessee; ^8^ Nicklaus Children’s Research Institute, Nicklaus Children’s Hospital Miami Florida; ^9^ National Human Genome Research Institute Bethesda Maryland

**Keywords:** attitude, common measures, genomic medicine, IGNITE, plan to share, quality of life

## Abstract

**Background:**

The IGNITE network funds six genomic medicine projects. Though interventions varied, we hypothesized that synergies across projects could be leveraged to better understand the participant experiences with genomic medicine interventions. Therefore, we performed cross‐network analyses to identify associations between participant demographics and attitudes toward the intervention (attitude), plan to share results (share), and quality of life (QOL).

**Methods:**

Data collection for demographics, attitude, share, and QOL surveys were standardized across projects. Recruitment and survey administration varied by each project's protocol.

**Results:**

Participants (*N* = 6,817) were 67.2% (*N* = 4,584) female, and 37.4% (*N* = 3,544) were minority. Mean age = 54.0 (sd 14.a). Younger participants were as follows: (1) more positive in attitude pre‐intervention (1.15‐fold decrease/10‐year age increase (OR)) and more negative after (1.14‐fold increase OR); (2) higher in QOL pre‐intervention (1.07‐fold increase OR) and postintervention; (3) more likely to share results (1.12‐fold increase OR). Race was significant when sharing results (white participants increased OR = 1.88), but not for change in QOL pre–postintervention or attitude.

**Conclusion:**

Our findings demonstrate the feasibility of this approach and identified a few key themes which are as follows: age was consistently significant across the three outcomes, whereas race had less of an impact than expected. However, these are only associations and thus warrant further study.

## 1 BACKGROUND

The emergence of technologies that permit rapid, low‐cost DNA sequencing has sparked the field of genomic medicine, “an emerging medical discipline that involves using genomic information about an individual as part of their clinical care and the health outcomes and policy implications of that clinical use.”(Manolio, 2016) Healthcare providers can use genomic information to help select safer and more effective medications (pharmacogenetics), identify individuals at risk for disease, improve the ability to predict disease onset/severity, and refine disease diagnosis. The rapid growth of the genomic medicine field has created an urgent need to identify best practices for implementing genomic interventions in healthcare systems and understanding factors that influence implementation success. The Implementing GeNomics In pracTicE (IGNITE) network was funded to identify effective ways to incorporate genomic information into clinical care and develop clinical decision‐making support for providers across diverse healthcare settings.(Weitzel et al., 2016).

The IGNITE network comprises six sites, each evaluating different genomic medicine interventions. These projects have been described in detail previously and, therefore, are summarized briefly here. (1) Duke University Medical Center (Duke FHH) conducted a study evaluating a web‐based, patient‐facing risk assessment program with integrated clinical decision support for primary care providers and patients at four geographically and culturally diverse health systems. The program, MeTree, allowed patients to enter personal and family health history data to generate clinical decision support for 27 conditions. The primary outcome was patient receipt of risk‐concordant care (Wu et al., 2015). (2) The University of Maryland (Maryland MODY) conducted a study to evaluate the impact of a multi‐pronged approach to identification, diagnosis, result disclosure, and therapy for individuals with highly penetrant genetic forms of diabetes, including maturity onset diabetes of the young (MODY) and neonatal diabetes. They also developed a MODY screening tool to help clinicians identify patients at risk for MODY. Primary outcomes included changes in diagnosis and treatment, clinical (glycemic control) and patient‐reported outcomes, cost‐effectiveness, and resource utilization. (3) The University of Florida (Florida PGx) conducted multiple pragmatic, proof‐of‐concept trials,(Cavallari et al., 2017) two relevant for cross‐network analyses were: a comparative effectiveness study in patients with gastroesophageal reflux disease who were randomized to either conventional or *CYP2C19*‐guided proton pump inhibitor therapy with a primary outcome of GERD control; and a nonrandomized cluster study to compare the change in pain intensity with *CYP2D6*‐guided vs. usual chronic pain management in patients taking select opioids. (4) The Icahn School of Medicine at Mount Sinai (Sinai APOL1) employed stakeholder engagement to conduct a randomized, controlled trial evaluating the impact of testing African ancestry adults with hypertension for *APOL1* renal risk variants. Results were returned to participants and their primary care providers. The primary outcome was hypertension control.(Horowitz et al., 2016) (5) Vanderbilt University Medical Center (Vanderbilt PGx) conducted a multisite trial integrating genetic data in EHRs at diverse sites to assess if it alters physician behavior toward a vision of individualized medicine.(Peterson et al., 2016) The trial established the processes, knowledgebase, and infrastructure necessary to disseminate clinical genetic testing, results reporting, and decision support by building on two clinical genotyping efforts at Vanderbilt which include, the Pharmacogenomic Resource for Enhanced Decisions In Care and Treatment (PREDICT) program(Pulley et al., 2012) which prospectively tests patients for “high‐value” germline pharmacogenomic variants and places these variants in the EHR; and the Personal Cancer Medicine Initiative (PCMI), which routinely performs multiplex tumor mutation testing.(Kusnoor et al., 2016) (6) The Indiana University School of Medicine (Indiana PGx) conducted a randomized clinical trial comparing outcomes between individuals receiving pharmacogenetic testing and no testing. Inpatient and outpatient participants newly prescribed one or more of 27 different medications with pharmacogenetic implications were followed up for 12 months. Intervention participants’ genotype results were incorporated in the EMR and providers were notified if actionable variants were identified. The primary outcomes were hospital and outpatient economic costs and adverse events over 1 year. Most, but not all of these IGNITE projects obtained primary outcomes from the EMR and all included patient surveys to obtain secondary and even tertiary outcomes.

Given the limited knowledge about implementation of genomic medicine, the IGNITE network sought to leverage these studies to identify common themes across genomic medicine interventions. To this end, the Common Measures Working Group (CMG) was developed to aid in identification of evidence that could guide genomic medicine implementation.(Orlando et al., 2018) Its mission is “to gather data, evaluate, and disseminate methods of genomic medicine implementation research across diverse projects conducted by IGNITE members.” To accomplish this objective, the CMG created a plan to identify constructs and associated measures pertinent to genomic medicine overall; standardize data collection across projects; combine data in a central database for cross‐network analyses; and develop a testable model for genomic medicine implementation research based on IGNITE research findings. By utilizing a searchable, centralized database, the network hoped to enhance diversity, increase statistical power, and improve external validity in comparison to what the individual sites could generate alone.

In this paper, we report cross‐network analyses of associations between patient demographic factors and three patient‐centered outcomes identified by the CMG as critical to understanding how genomic medicine interventions impact patients include: plan to share results, attitudes toward the intervention to which they were exposed, and quality of life. Studies on demographic differences in genomic medicine studies thus far have focused on issues related to study participation. For example, in a national sample of African Americans, female gender and distrust were associated with lower intentions of participating in a hypothetical precision medicine study. In addition, African Americans were less likely to provide a sample than White people, though this difference was not significant after controlling for trust.(Halbert, McDonald, Vadaparampil, Rice, & Jefferson, 2016) In a survey of individuals from 11 US healthcare systems, willingness to participate in a biobank project was associated with self‐identified White race, higher education, lower religiosity, perception of research benefits, fewer concerns, and fewer informational needs.(Sanderson et al., 2017) Our cross‐network analyses extend this previous research by examining the demographic differences in perceptions of personal utility in the context of genomic medicine studies.

## 2 METHODS

### 2.1 Study design

For full details of each IGNITE project's study design, see their published protocols. Details relevant to data collected for the cross‐network analysis are presented herein. All six projects agreed to administer cross‐network survey questions, pre‐ and post‐study intervention, where feasible, in addition to their other data collection. All patients were consented for the survey. Methods of administration varied at each site. Three projects (Duke, Florida PGx, and Indiana PGx (for all but one recruiting location)) used a web‐based survey administered to patients via REDCap (Harris et al., 2009) or Qualtrics at baseline, and two projects (Duke FHH and Florida PGx) at 3 months postintervention. One project verbally collected responses from patients and entered them into REDCap at baseline (Sinai APOL1) and two projects at 3 months postintervention (Sinai APOL1 and Florida PGx). Two projects collected responses via a written questionnaire, and data were entered into REDCap by study personnel at baseline (Maryland MODY, Indiana PGx at one recruiting location) and one project (Maryland MODY) at 18 months postintervention. One project (Vanderbilt PGx) did not recruit patients and therefore could not administer a patient survey or contribute data to this cross‐network analysis. Table 1 shows the number of individuals at each project, and in total, on whom data were gathered for each measure. Cross‐network analyses were only performed when at least two projects contributed data.

**Table 1:** Summary of Patient demographics and survey data availability by project

Question	Timing	Duke‐FHH *N*	Florida PGx *N*	Indiana PGx *N*	Maryland MODY *N*	Sinai‐APOL1 *N*	Total
Age	Baseline	2,513	605	1,320	184	2053	6,675
Gender	Baseline	2,513	605	1,320	326	2053	6,817
Ethnicity	Baseline	1,532	557	1,303	319	2053	5,764
Race	Baseline	2,021	602	1,272	325	1999	6,219
Education	Baseline	2,454	600	0	313	2049	5,416
Attitude	Baseline	0	605	1,328	0	2048	3,981
Attitude	Postintervention	0	117	0	0	1925	2,042
Plan to share results	Baseline	0	605	1,315	180	2053	4,153
Plan to share results	Postintervention	0	0	0	0	2056	2,056
Quality of life	Baseline	2,441	601	0	180	2053	5,275
Quality of life	Postintervention	1,122	0	0	0	1925	3,047

Data in the projects’ REDCap and Qualtrics databases were standardized using a commonly agreed upon format for each data element and transformed into a formatted Excel file prior to sending to the network coordinating center. Data were cleaned using python scripts and organized for import into the MS SQL relational database with SQL's database import tools. Quality control checks included both manual verifications of the data and programmatic (MS SQL scripts) validation queries.

### 2.2 Ethical compliance

All projects were approved by the IRBs at the involved health systems.

### 2.3 Populations

The populations differed across projects. Duke FHH recruited from primary care populations at five national healthcare systems with different patient populations; Sinai APOL1 from African Americans attending primary care clinics in Harlem and the Bronx, NY; Indiana PGx from in‐ and out‐patients who received a prescription for a relevant medication; Vanderbilt PGx from the health system; Florida PGx from primary care and gastroenterology clinics in the Florida Health system; and Maryland MODY recruited from endocrine clinics and the community.

### 2.4 Measures

The three variables of central interest were attitude toward the intervention, plan to share results, and quality of life using the SF‐1.(Busija et al., 2011) The first two were developed by the IGNITE network as part of the CMG's goal to capture data representing important constructs in the Consolidated Framework for Implementation Research (see published paper for detailed description of the process).(Orlando et al., 2018) Specifically, the attitude question asks, “It is a good idea to [insert your genomic intervention]] to find out whether [insert outcome of your genomic intervention]?” with responses ranging from *strongly agree* to *strongly disagree* on a 5‐point Likert scale. The plan to share results question asks, “Do you plan to share [test] results with anyone?” Response options are as follows (check all that apply): *no*, *yes with family members* (*spouse/partner, parents, children, brothers/sisters, other* (fill in the blank)), *yes with friends*, *yes with healthcare professionals*, *yes with other* (fill in the blank), or *unsure*. The SF‐1 quality of life response options were as follows: *excellent*, *very good*, *good*, *fair*, and *poor*. These three variables were examined by demographic factors, including age, gender, race, ethnicity, and education level.

As previously described, not all projects were able to collect all variables, and not all projects were able to collect variables in the same way. Those collected in different ways (e.g., different response choices) were mapped to a harmonized set of responses. For example, education level, which was captured using different response options across projects, was remapped and harmonized into four mutually exclusive levels (e.g., *less than 8 years*, *8 through 11 years*, *12 years or completed high school*, and *high school or less* were collapsed to *high school or less*). In addition, plan to share results was collapsed into yes/no categories as follows: If any of the plan included one of the *yes* responses, then it was categorized as yes; *no* responses remained no; and all *unknown*, *unsure*, or unreported responses were excluded from analyses.

### 2.5 Statistical analysis

Demographics and baseline and postintervention survey responses were summarized using counts and percentages or means and standard deviations. Differences in patients’ survey responses (attitude toward intervention, plan to share results, and quality of life) by patients’ demographics were assessed using ordinal or logistic regression. Significance of each demographic feature was assessed using the likelihood ratio test of nested models and the significance threshold set at (unadjusted) *p*≤0.05. Categorical predictors that had more than two levels (e.g., race, education level) and were associated with participants’ survey responses were further evaluated for differences among all pairwise combinations using generalized linear hypothesis testing. Pairwise comparisons were adjusted for multiple testing using the Benjamini–Hochberg multiple testing correction.

Changes in participants’ survey responses from baseline to postintervention were quantified only for questions for which two or more different demonstration projects provided both baseline and postintervention survey responses. Counts and percentages were used to describe change/no change and direction of changes in response. Shifts in mean response from baseline to post were assessed using the Wilcoxon signed‐rank test. The magnitude of change from baseline to post, that is, the number of steps up or down on the response scale was quantified for each participant. Bivariate analyses of independence between magnitude of change and participants’ demographics were assessed using Pearson's chi‐squared tests or ANOVA *F*‐tests. Ordinal regression was used to model participants’ magnitude of change as a function of demographics and project. The significance of each independent variable was assessed using likelihood ratio tests of nested models and the significance threshold set at (unadjusted) *p*≤0.05. All statistical analyses were completed using r.(R Development Core Team, 2011).

## 3 RESULTS

Descriptive statistics for participants in each project and in total are presented in Table 2. Overall, as is common in research, participants were predominantly female (66.9%) and middle‐aged (mean 54.6, sd 13.3). Age was differed with project. This difference was driven by Maryland MODY whose project was to identify those with highly penetrant genetic forms of diabetes, primarily recruited those with diabetes at a young age. The mean age of participants at Maryland MODY was 10–18 years younger than the mean age in other projects. Race, ethnicity, and education were also significantly different between projects. These differences were primarily driven by Sinai APOL1 which recruited only African ancestry adults. There were a predominantly low resource and low literacy population, who were significantly different from other project populations.

**Table 2:** Descriptive statistics of participants from each site for each project and in total

	Duke‐FHH *N* (%)	Florida PGx *N* (%)	Indiana PGx *N* (%)	Maryland MODY *N* (%)	Sinai‐APOL1 *N* (%)	Total *N* (%)
Gender						
Male	791 (31.5)	203 (33.6)	428 (32.4)	119 (36.5)	692 (33.7)	2,233 (32.8)
Female	1722 (68.5)	402 (66.5)	892 (67.6)	207 (63.5)	1,361 (66.3)	4,584 (67.2)
Missing/Not reported	1	0	10	0	0	11
Age* mean (sd)	57.0 (14.1)	58.0 (13.7)	49.9 (14.4)	40.4 (17.4)	53.1 (9.9)	54.0 (13.6)
Ethnicity*						
Hispanic	41 (2.68)	34 (6.1)	40 (3.12)	6 (1.88)	117 (5.7)	238 (4.15)
Non‐Hispanic	1,491 (97.3)	523 (93.9)	1,240 (96.9)	313 (98.1)	1936 (94.3)	5,503 (95.9)
Missing/Not reported	982	48	50	7	0	1,087
Race*						
African American	165 (8.2)	109 (18.1)	448 (35.2)	81 (24.9)	1866 (93.3)	2,669 (42.9)
American Indian or Native Alaskan	3 (0.2)	10 (1.7)	4 (0.3)	1 (0.3)	9 (0.5)	27 (0.4)
Asian	29 (1.43)	6 (1)	10 (0.79)	15 (4.62)	0 (0)	60 (1.0)
Mutiracial	48 (2.4)	9 (1.5)	0 (0)	6 (1.9)	123 (6.2)	186 (3.0)
Native Hawaiian or other Pacific Islander	0 (0)	1 (0.17)	3 (0.24)	0 (0)	0 (0)	4 (0.06)
White participants	1776 (87.9)	467 (77.6)	807 (63.4)	222 (68.3)	1 (0.1)	3,273 (52.6)
Missing/Not reported	493	3	58	1	54	609
Education*						
High School or Less	235 (9.6)	220 (36.7)	0 (0)	112 (35.8)	876 (42.8)	1,443 (26.6)
Some College, Vocational, or Technical training	391 (15.9)	206 (34.3)	0 (0)	53 (16.9)	609 (29.7)	1,259 (23.4)
College Graduate	709 (28.9)	104 (17.3)	0 (0)	71 (22.7)	564 (27.5)	1,448 (26.7)
Postgraduate	1,119 (45.6)	70 (11.7)	0 (0)	77 (24.6)	0 (0)	1,266 (23.5)
Missing/Not reported	60	5	1,330	13	4	715

* Significantly different by project.

**Figure 1:**
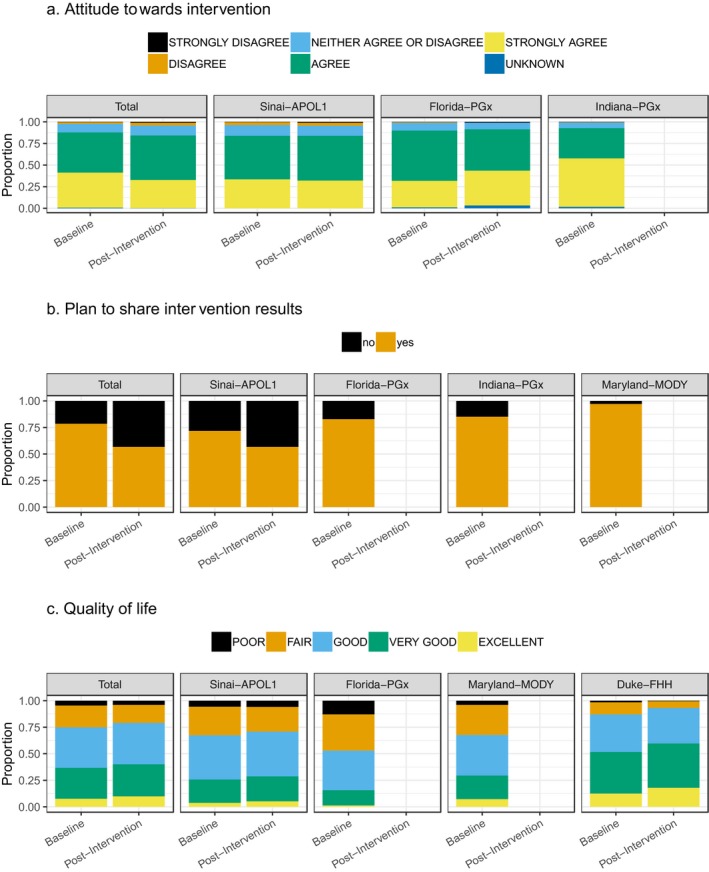
Proportion of participant's responses to survey questions for each project and in total. (a) Attitude towards intervention. (b) Plan to share intervention results. (c) Quality of life.

Below and in Figure 1 we report the results of analyses for each patient survey question: attitude toward the genomic medicine intervention, plan to share results, and quality of life.

### 3.1 Attitude toward the genomic medicine intervention

Three projects provided data on participant attitudes toward the genomic intervention at baseline and two at postintervention. At baseline, a mean of 87.0% of participants indicated they agreed or strongly agreed that the intervention would be beneficial, 9.9% were neutral, and 2.3% disagreed or strongly disagreed. There were significant differences in response by project, both when responses were collapsed to three levels and with the original 5‐point Likert scale. Specifically, Indiana PGx differed from other sites at the “strongly agree” (Indiana PGx 56.0% vs. 30.6% and 33.5%) and “agree” (Indiana PGx 35.1% vs. 58.1% and 50.3%) response levels. Two multivariable models were analyzed—one including education as an explanatory variable that excluded Indiana PGx data (since they did not collect education level) and the other exclude education that incorporated Indiana PGx data. In the former, gender (females>males, OR =1.22, 95% CI 1.04–1.43), education level (college graduate significantly higher than high school or less, OR =1.30, 95% CI 1.07–1.57), and age (1.15‐fold increase in odds of a more positive attitude per 10‐year reduction in age) were significantly associated with attitude while project, ethnicity, and race were not. In the latter, the significant variables changed to project (Indiana PGx had significantly higher attitudes than Florida PGx and Sinai APOL1), gender (females>males, OR = 1.33, 95% CI 1.16–1.51), and age (1.18‐fold increase in odds of a more positive attitude per 10‐year decrease in age). Only age was significant in both models.

Postintervention, the mean percentage of agree/strongly agree was 84.9%, 11.5% were neutral, and 4.2% disagreed or strongly disagreed. In multivariable modeling, only age was significant (1.14‐fold increase in odds of a higher attitude per 10‐year decrease in age).

There were 2037 paired baseline to postintervention responses for two projects. Of these, 55.9% stayed the same and 44.1% changed. Among the respondents that changed, 48.1% had a more positive attitude after the intervention than before, while 51.9% had a more negative attitude after the intervention. In multivariate modeling of change magnitude, no variables were significant.

### 3.2 Plan to share results

Four projects provided plan to share data at baseline and only one on postintervention; therefore, analyses were performed only on baseline responses. Uniformly, participants indicated that they have planned to share their results (mean 78.52%, range 71.8%–97.1%). The project most likely to share was Maryland MODY at 97.1%. In multivariate modeling, only project and age (1.12‐fold increase in odds of sharing per 10‐year increase in age) were statistically significant. In pairwise comparisons, Maryland MODY had significantly higher odds of sharing than both Sinai APOL1 (OR = 8.93, 95% CI 2.67–29.9) and Florida PGx (OR = 7.13, 95% CI 2.13–23.8). In multivariate modeling that excluded education and, thus, included Indiana PGx data, project and race were statistically significant. Pairwise comparisons revealed all project comparisons, except for Sinai APOL1 and Florida PGx, were significantly different (Maryland MODY>Indiana PGx OR = 5.78, 95% CI 1.80–18.6; Indiana PGx>Florida PGx OR =1.39, 95% CI 1.04–1.86; Florida PGx =Sinai APOL1 OR =1.12, 95% CI 0.81–1.54). White participants were more likely to share results than African American (OR = 1.88, 95% CI 1.42–2.49) and multiracial participants (OR = 2.04, 95% CI 1.27–3.27).

### 3.3 Quality of fife

Four projects provided data on quality of life at baseline and two projects on quality of life postintervention. At baseline, 36.8% reported excellent or very good quality of life, 38.1% good, and 25.1% fair or poor. There were significant differences by project, with Florida PGx having a much higher percentage of participants reporting poor quality of life (12.9%) vs. Duke FHH (1.5%), Maryland MODY (3.9%), and Sinai APOL1 (5.6%). In multivariable modeling, project, ethnicity (non‐Hispanic were 1.60‐fold [95% CI 1.16–2.22] more likely to have a higher level than Hispanic), race (with White participants having 1.82‐, 4.23‐, and 1.59‐fold increase in odds of a higher quality of life than African Americans, American Indians, and multiracial participants, respectively), education (postgraduate>college graduate [OR_postgrad vs. college grad_= 1.67, 95% CI 1.39–2.01]>some college [OR_college grad vs. some college_ = 1.61, 95% CI 1.39–1.89]> high school or less [OR_some college vs. high school or less_ = 1.17, 95% CI 1.00–1.37]), and age (1.09‐fold increase in odds of higher quality of life per 10‐year increase in age) were all statistically significant.

Postintervention, only Duke FHH and Sinai APOL1 collected quality of life. Overall 40.1% of participants reported excellent or very good quality of life, 39.0% good, and 20.9% fair or poor (*p* < 0.05 for Duke FHH vs. Sinai APOL1). Notably, Florida PGx had a significantly larger percentage of participants with “poor” quality of life than the other projects. In multivariable modeling, gender (females <males, OR 0.77, 95% CI =0.66–0.89), education (postgraduate>college graduate [OR_postgrad vs. college grad_= 1.65, 95% CI 1.26–2.14]>some college [OR_college grad vs. some college_ =1.40, 95% CI 1.16–1.71]> high school or less [OR_some college vs. high school or less_ = 1.20, 95% CI 0.99–1.44]), and age (1.07‐fold increase in odds of higher quality of life per 10‐year increase in age) were statistically significant.

There were 3,040 paired survey responses at Duke FHH and Sinai APOL1 for a baseline vs. postintervention quality of life analysis (Table 3). Of these, 60.4% had no change in quality of life after the intervention, while 39.6% did (*p* < 0.05). Of those who had a change in quality of life, 57.1% had an improvement and 42.9% had a decrease 3 months after the intervention. In multivariable modeling, only age was statistically significant, with older participants being more likely to have a decline in quality of life (1.11‐fold increase in odds of decreased quality of life per 10‐year increase in age).

**Table 3:** Change in quality of life and attitudes toward the genomic medicine intervention pre and post intervention

A) Quality of Life						
Baseline (N)	Post–Intervention (N)					
	POOR	FAIR	GOOD	VERY GOOD	EXCELLENT	NA
POOR	**51**	50	7	6	0	123
FAIR	51	**299**	204	34	7	491
GOOD	13	148	**745**	261	21	825
VERY GOOD	3	21	218	**565**	97	634
EXCELLENT	0	1	10	52	**176**	164
NA	0	0	4	3	0	849

B) Attitude toward the genomic medicine intervention							
Baseline (N)	Post–Intervention (N)						
	STRONGLY DISAGREE	DISAGREE	NEITHER AGREE OR DISAGREE	AGREE	STRONGLY AGREE	UNKNOWN	NA
STRONGLY DISAGREE	**2**	0	3	1	1	0	6
DISAGREE	4	**13**	19	21	6	0	16
NEITHER AGREE OR DISAGREE	1	19	**69**	121	32	0	152
AGREE	10	20	113	**656**	227	3	821
STRONGLY AGREE	4	13	30	251	**396**	2	920
UNKNOWN	0	0	0	0	0	0	31
NA	0	0	0	4	1	0	2,842

Note

Cells are shaded by decreased quality of life or attitude (dark gray), increased quality of life or attitude (light gray), or no change (unshaded, bold text) 3 months after the intervention. For quality of life projects with pre‐post data are Duke FHH and Sinai APOL1 and for Attitudes the projects are Florida PGx and Sinai APOL1.

## 4 DISCUSSION

To date, there have been few studies examining the impact of genomic medicine interventions in real‐world environments.(Gaff et al., 2017; Orchard, 2015) This paper leverages data from six unique genomic medicine projects to explore the broader impact of genomic medicine interventions on participants in three key areas: attitudes about the intervention, plan to share results, and quality of life. While combining data from different studies is challenging, being part of a network, such as IGNITE, affords the opportunity to work in concert to harmonize data and consider cross‐network outcomes.

Our findings demonstrate the feasibility of this approach and identified a few key themes which include: age was consistently significant across the three outcomes, whereas race had less of an impact than expected. Notably, younger participants had a more positive attitude toward genomic medicine interventions than older participants before the intervention, but were more likely to have a decline in their attitude after the intervention. They were also more likely to have a higher quality of life before the intervention and have an increase after the intervention than older adults. Lastly, younger people were more likely to share results. In a 2013 study of public attitudes about genomics that was dominated by those aged 18–29, attitudes were much more positive than in previous studies in European populations (Haga et al., 2013); however, there are no studies assessing change in attitude or quality of life after a genomic medicine intervention. While just associations, these are encouraging findings for several reasons, including risk‐based genomic medicine interventions are more likely to benefit younger individuals and younger individuals are coming of age in a more genomically informed era and may be adopting a more optimistic view of genomic medicine in general.

What was also striking in our findings was the absence of race as a significant variable in most of the multivariate models. Race was (1) not associated with attitude toward the intervention in any of the models, (2) associated with sharing results when education was removed from the model, and (3) associated with baseline quality of life (as is well known), but not associated with change in quality of life after the intervention. While interesting, these are only associations, not causal relationships, and given the differences in study interventions, populations, and designs we are limited to the conclusions that we draw.

Prior to the completion of the Human Genome Project, general knowledge about genomics was limited. Since that time, several initiatives to educate the public of the potential value have been undertaken.(Dressler, Jones, Markey, Byerly, & Roberts, 2014) Still, public attitudes on genomic testing were quite diverse and varied by age, education, gender, race, and ethnicity(Aro et al., 1997; Vermeulen, Henneman, van El, & Cornel, 2014), suggesting that educational initiatives may need to be tailored to the populations they are designed to impact. With the more recent advent of direct‐to‐consumer genetic testing, multimedia and social networking advertising campaigns have increased knowledge and interest in genomics.(Bowen & Muin, 2018) The uniformly strong positive attitude toward the value of genomic interventions in IGNITE may be related to these more recent campaigns. Notably, statistically significant differences in attitudes continue to exist at baseline based on age, gender and level of education or project and age (if education is excluded from the model), but only age was associated with a change in attitude after the intervention. This is an interesting finding, not previously reported, that warrants further investigation. The conflicting role of education (depending upon the model) suggests that there may have been some improvement in this area with the recent social‐media campaigns since recent studies have shown that social‐media adoption is high, even in lower income, lower education groups (pew research center http://www.pewinternet.org/2018/03/01/social-media-use-in-2018/). This finding also warrants further investigation. Consistent with the generally positive attitude toward the genomic interventions, respondents also reported a strong willingness to share their genomic information with others, though race continues to play a significant role.

While these findings are intriguing, there are a number of limitations to this cross‐network analysis. First, the surveys did not link a change in quality of life or attitude to the actual genomic intervention. Therefore, these findings only describe associations rather than cause and effect. Second, the interventions and methods employed to gather data differed across projects. We attempt to assess the impact of these differences by accounting for the “project” each participant enrolled in as part of the statistical models, but other confounders make adjustment difficult. For example, all participants in Sinai's study had African ancestry and most had limited education. Thus, caution must be employed when interpreting the results and applying our findings to broader populations. Of course, we also know that individuals who enroll in research studies are different than those who do not, which may impose a positive bias on our findings. Given these limitations, we do not suggest that our findings are conclusive, but, rather, that there seem to be interesting (and positive) trends that warrant further investigation.

This study highlights the feasibility of merging data from different genomic medicine studies within a network, even when the study's each have a different intervention. There were significant challenges with implementing surveys at all sites and in some cases study design differences (such as not consenting patients) that limited some of the data available for cross‐network analyses; however, with early planning, shared goals, and standardization of measures it is achievable. The benefit is the ability to look at the same variable (e.g., QOL) from a variety of perspectives and across a variety of populations.

In summary, although the IGNITE Network comprises different patient populations and genomic medicine interventions, we identified several common themes in our analysis. Importantly, we highlight how the demographics of genomic medicine participants affect patient‐centered outcomes. The identified associations highlight areas and populations that may need more focus and additional strategies to increase positive perceptions and study participation. Although we faced a number of challenges, this paper can serve as a guide to networks or other projects that would like to enhance statistical rigor or explore previously unconsidered outcomes by combining data across studies.

## CONFLICT OF INTEREST

None of the authors have any conflicts of interest to disclose. This work was funded by the NIH grants cited in the manuscript.
